# Recent Advances in FIB-SEM for Microstructural Characterization of Metallic Materials

**DOI:** 10.3390/ma19091818

**Published:** 2026-04-29

**Authors:** Yi Qiao, Yong Zhang

**Affiliations:** State Key Laboratory for Advanced Metals and Materials, University of Science and Technology Beijing, Beijing 100083, China

**Keywords:** focused ion beam—scanning electron microscopy (FIB-SEM), multimodal correlative characterization, metallic material characterization

## Abstract

Since its introduction, focused ion beam (FIB) technology has expanded from micro/nanofabrication in the semiconductor industry to the field of multimodal characterization of metallic material microstructures. This article systematically reviews the latest research advances in FIB-SEM technology in the field of metallic materials science. The fundamental principles and system functions of FIB-SEM are introduced, with an emphasis on its key applications in two-dimensional and three-dimensional morphological characterization, as well as specimen preparation for transmission electron microscopy (TEM) and atom probe tomography (APT). The combined strategies of FIB-SEM with electron backscatter diffraction (EBSD), time-of-flight secondary ion mass spectrometry (TOF-SIMS), and other characterization techniques are also discussed. Current developments indicate that FIB-SEM technology is advancing toward multi-ion-source synergy and multimodal integration. In the future, combined with artificial intelligence and big data analysis, it is expected to enable high-throughput, correlative measurements of multidimensional properties at the micro scale, providing important technical support for “materials genome” research in metallic materials.

## 1. Introduction

Since the advent of the FIB-SEM dual-beam system (1988, P. Sudraud and G. Ben Assayag) [1], the combined system of a focused ion beam (FIB) and scanning electron microscope (SEM)—the FIB-SEM dual-beam system—has become the mainstream platform for FIB technology applications. In this system, the SEM and FIB can both be focused on the same region of interest on a material: the SEM provides imaging and positioning while the FIB performs ion-beam machining. The application of FIB has thus expanded beyond semiconductors to materials science, geology, biology, and other fields, where it has flourished [2,3,4].

## 2. Principles of FIB-SEM

### 2.1. FIB

A typical FIB instrument comprises a vacuum system and chamber, ion source, ion-optical column, specimen stage, detectors, gas injection system, and control system [5]. The ion source is the key component of any FIB system; the liquid metal ion source using gallium (Ga) was the origin of practical FIB technology. Modern FIB systems have since been developed using a variety of ion species, including helium (He), neon (Ne), gallium (Ga), argon (Ar), and xenon (Xe). Based on the ion-source operating principle, FIB systems can be classified into liquid metal ion source (LMIS) systems, gas field ionization source (GFIS) systems, and plasma ion source systems, as shown in Table 1.

Liquid Metal Ion Source (LMIS) [6,7,8,9]: Operating principle—field evaporation of a liquid metal. Ion types—metal ions (Ga^+^, Bi^+^). Beam spot size—relatively small (~5 nm). Beam current—moderate to high (10^−3^–10^2^ nA). Typical applications—general-purpose FIB machining, circuit editing, TEM specimen preparation, and nanoscale deposition. Nowadays, Ga ions are the mainstream ion source in current focused ion beam systems due to their high processing precision, relatively high efficiency, and low processing damage.

Gas Field Ionization Source (GFIS) [10,11,12,13]: Operating principle—field ionization of a gas-phase atom. Ion types—gas ions (He^+^, Ne^+^). Beam spot size—extremely small (<0.5 nm). Beam current—relatively low (10^0^–10^2^ pA). Typical applications—helium/neon ion microscopy for ultra-high-resolution imaging and nanoscale patterning. Due to the small size of He and Ne ions, they are very prone to implantation into materials. Currently, researchers rarely use these ion sources for processing; instead, they often take advantage of their ion channeling effect to perform morphological observation at low voltages (ion implantation is closely related to the initial ion energy).

Plasma Ion Source [14,15,16]: Operating principle—plasma generated by gas discharge. Ion types—gas ions (Xe^+^, Ar^+^). Beam spot size—relatively large (>10 nm). Beam current—large (10^2^–10^3^ nA). Typical applications—rapid, high-throughput specimen preparation and deep etching (e.g., three-dimensional tomography).

### 2.2. FIB-SEM

**Figure 1:**
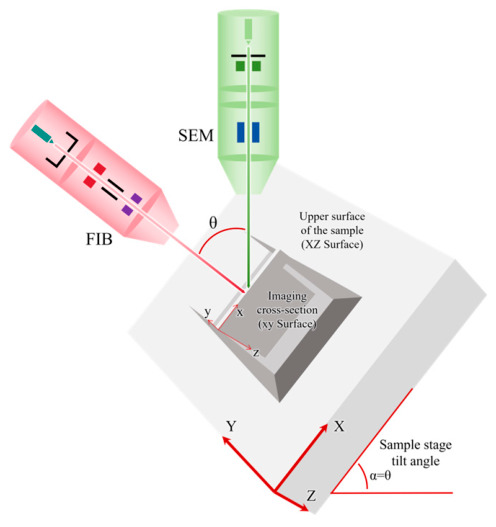
Schematic of FIB-SEM specimen processing.

The FIB-SEM system integrates the FIB and SEM into a single vacuum chamber, combining nanoscale machining (FIB) with high-resolution imaging (SEM). In an FIB-SEM system, the FIB ion column and the SEM electron column are typically arranged at an angle of 36–38° to each other. During operation, the FIB ion column is usually oriented perpendicular to the material being processed or characterized, as shown in Figure 1. The specimen stage of an FIB instrument is a precision multi-degree-of-freedom motion system. Its core motion capabilities include standard X, Y, and Z orthogonal linear translations (forming a Cartesian coordinate system), together with integrated rotational and tilt degrees of freedom for complex attitude adjustment.

**Figure 2:**
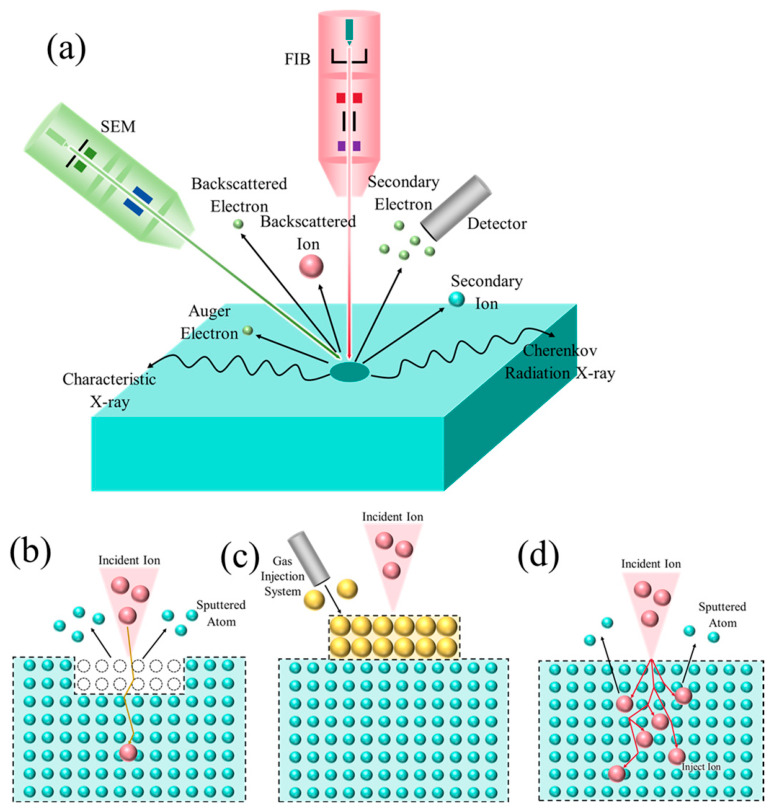
Schematic of the basic functions of a focused ion beam system: (**a**) scanning imaging; (**b**) sputter etching; (**c**) induced deposition; (**d**) ion implantation.

The interaction behaviors between FIB-SEM and material atoms can be divided into those of the ions from the FIB column and those of the electrons from the SEM column, as shown in Figure 2a.

Ion–atom interactions in FIB: Incident ions undergo elastic and inelastic collisions with atoms in the material, producing elastic and inelastic scattering, respectively. Elastic collisions transfer energy from the incident particle to material atoms, triggering a cascade of secondary collisions. Some incident ions are backscattered at the material surface and leave as backscattered ions. Other incident ions, through the collision cascade, knock material atoms from their lattice sites toward the surface, creating recoil atoms. When a recoil atom has sufficient energy to overcome the surface binding energy, it is ejected from the material as a sputtered particle. Some charged sputtered particles become secondary ions (SIs) after further ionization; these signals can be collected by a detector and used for imaging. The inelastic collision channel also excites plasmons and phonons while producing a large number of secondary electrons (SE), whose signals can likewise be collected and used to form images.

Electron–atom interactions in SEM: Incident electrons undergo elastic and inelastic scattering with material atoms. Elastic scattering causes the large-angle deflection of electrons, generating backscattered electrons (BSE) whose yield is related to atomic number and can be used for compositional analysis. Backscattered electrons originating from a certain depth within a crystalline material, when satisfying the Bragg diffraction condition, produce characteristic Kikuchi diffraction patterns that can be used to analyze the crystal orientation, phase, and strain (EBSD). Inelastic scattering transfers energy to the specimen, producing multiple signals: the excitation of low-energy secondary electrons from the near-surface region forms the basis of morphological imaging (SE image); the excitation of inner-shell electrons generates characteristic X-rays for elemental analysis (EDS); the Auger electrons and cathodoluminescence are also produced. These signals originate from different depths within the specimen surface and together constitute the multifunctional SEM platform for high-resolution imaging, micro-area compositional analysis, and crystal structure and orientation analysis.

When a focused ion beam scans the specimen surface, it excites secondary electron (SE) and secondary ion (SI) signals that can be collected by various detectors equipped within the FIB system. Most FIB systems are equipped with a gas injection system; combined with SiO_2_, Pt, Au, W, and C gas precursors, they can realize ion-beam-assisted induced deposition for material surface protection and nano-device assembly. Combined with XeF_2_ gas, the assisted etching of silicon is possible; combined with H_2_O gas, assisted diamond etching can be performed. Consequently, the functions of a focused ion beam system include ion exposure, ion implantation, ion etching, elemental deposition, integrated circuit fabrication, and mask repair [14,17,18,19,20], as shown in Figure 2.

**Figure 3:**
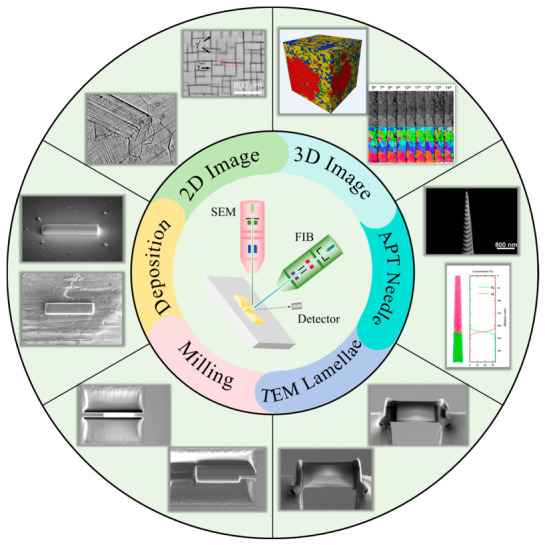
Examples of FIB-SEM micro-/nanofabrication processes and applications.

**Figure 4:**
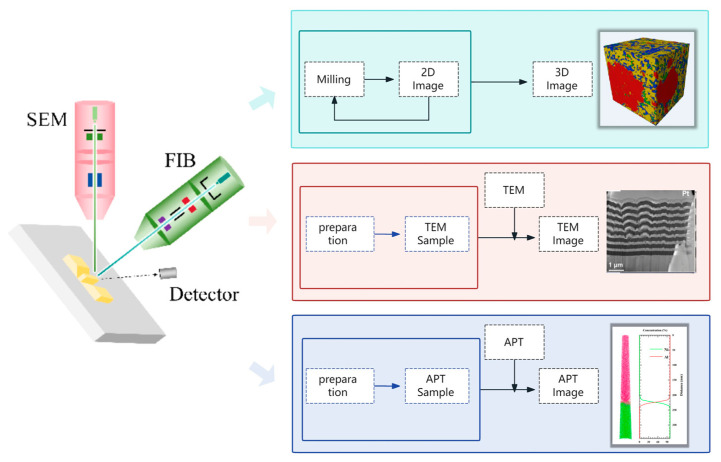
Flowcharts of three typical applications of FIB-SEM.

Based on the above interactions of particles with material atoms in both FIB and SEM, the functions of FIB-SEM can be summarized to include morphological observation, sputter etching, induced deposition, and ion implantation [14,21,22,23]. The schematic principle diagram is shown in Figure 2, and representative application examples are shown in Figure 3. Figure 4 shows the flowcharts of three typical applications, the details of which will be introduced in Section 3 and Section 4.

## 3. Applications of FIB-SEM in Morphological Characterization of Metallic Materials

### 3.1. Two-Dimensional Morphological Characterization

Due to the etching effect of the high-energy ion beam, the ion beam causes specimen damage while collecting signals; the extent of this damage depends on the energy and intensity (accelerating voltage and beam current) of the ion beam [23,24,25]. Therefore, continuous imaging using the ion beam as the signal source is generally avoided. However, the GFIS-based helium/neon ion microscope [14,15,16], which operates based on the quantum-mechanical tunneling effect, can be used for ultra-high-resolution imaging because of its low ion energy and minimal damage; its imaging contrast and surface detail are superior to those achieved by electron-beam imaging [17]. This is attributable to an extremely short de Broglie wavelength and a highly localized interaction volume, allowing ultimate surface imaging at sub-nanometer resolutions [15] and clearly revealing true atomic-scale surface steps and morphologies with almost no subsurface information interference. Hlawacek et al. [16] used a helium ion microscope to study silver alloys confined to Pt(111) surfaces, directly visualizing single-atomic-layer-high steps between terraces and measuring the periodicity of hcp/fcc patterns formed in two-to-three-layer-thick Ag/Pt alloy films. Wang et al. [17] found that even when HIM operates with a high voltage and low beam current, the fine details of particles in the image remain clearly visible, achieving a resolution superior to that of low-voltage high-beam-current SEM. This is because the secondary electrons excited by backscattered ions are much fewer than those excited by incident ions, thereby enhancing the image. For insulating materials, when HIM is operated at 20 kV and SEM at 5 kV respectively, HIM also exhibits better material contrast. This is attributed to the shallow interaction depth of He^+^ with the sample and the weak backscattered ion signal, making HIM sensitive to surface materials and enabling clearer differentiation between different materials.

In addition, the strong interaction between ions and atomic nuclei endows ion-beam images with excellent material and grain contrast, allowing the clear differentiation of grains with different orientations in polycrystalline materials and light-element components in composite materials. The positively charged incident ions naturally neutralize the surface charge of the specimen, enabling the direct, ultra-high-resolution imaging of insulators (e.g., ceramics, polymers, biological specimens) without prior conductive coating. Furthermore, the technique offers an exceptionally large depth of field and is free from edge-brightening artifacts, providing a more accurate representation of the three-dimensional geometry. These characteristics make the helium/neon ion microscope an indispensable and powerful tool for exploring true surface structures at the nanoscale, especially the surface properties of insulating materials.

On the other hand, it should be noted that focused helium ions can cause both amorphous damage and helium bubble damage in materials, and these types of damage are closely related to the helium ion beam fluence and the material properties. Taking Si and Cu as examples, Livengood et al. [26] found that, at helium ion beam energy of 32 keV and a current of 7.6 pA, an amorphous transition occurred at fluence of 5 × 10^16^ ions/cm^2^. When the fluence reached 1.6 × 10^17^ ions/cm^2^, helium bubbles with diameters of up to 3 nm appeared. At fluence of 5 × 10^17^ ions/cm^2^, the diameters of the helium bubbles reached 30 nm. In 4H-SiC, Song et al. [27] reported that the threshold fluence for amorphization and helium bubble formation is 1 × 10^17^ ions/cm^2^.

Therefore, when using a helium ion microscope for observation, reasonable control of the imaging time and ion beam fluence is an important strategy for researchers to avoid characterization damage. Exploring the helium ion characterization damage data for different materials, establishing a machine learning database, and expanding the intelligent capabilities of helium ion characterization are also important future research directions.

### 3.2. Three-Dimensional Morphological Characterization

**Figure 5:**
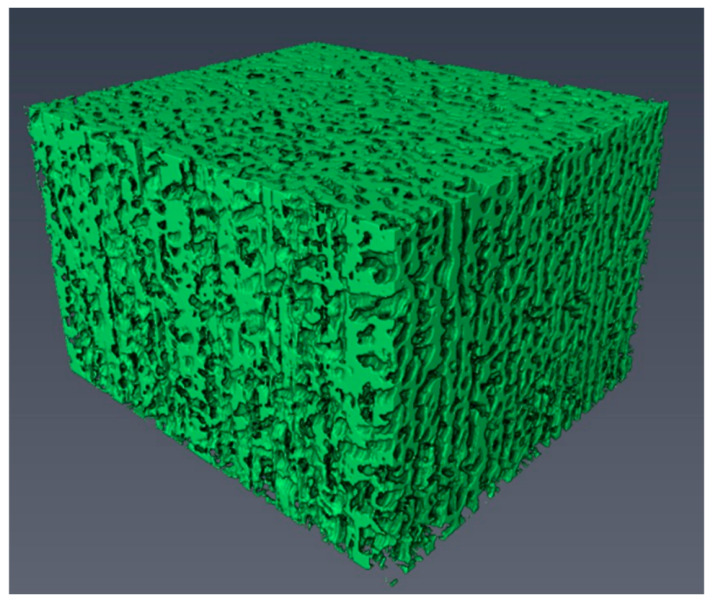
Three-dimensional imaging of nanoporous silver.

FIB-SEM three-dimensional tomography (FIB-SEM tomography) has gradually become a core tool for resolving the three-dimensional spatial structures of metallic materials, owing to its unique capability to acquire true three-dimensional information from moderate microvolumes (10–1000 μm^3^) at a nanometer resolution [28,29,30,31]. In 2004, HOLZER et al. [29] successfully reconstructed the three-dimensional structure of BaTiO_3_. Qiao et al. also employed an FIB-SEM dual-beam system to achieve slice-and-view 3D tomography, acquiring true three-dimensional (3D) information from moderate analysis volumes (10–1000 μm^3^) of a given material at a nanometer resolution, as shown in Figure 5. To resolve material structures, researchers often require that each structural unit be represented by at least nine pixels when acquiring images. If a large field of view is also needed, the acquisition of a single image takes a considerable amount of time. Considering sample stability, a typical acquisition time of 10 to 48 h is currently a common choice. Therefore, when observing nanoscale structures, 3D reconstruction is often limited to the micrometer scale. However, with advances in FIB-SEM autofocus, tracking techniques, data algorithms, and data storage, future 3D morphological characterization is trending toward high throughput and long-duration acquisition.

## 4. Applications of FIB-SEM in the Micro/Nanofabrication of Metallic Materials

Researchers have used FIB-SEM dual-beam systems, with real-time SEM monitoring, to precisely control key parameters such as the ion species, energy, beam current, and dwell time, successfully achieving the highly accurate, three-dimensional controllable fabrication of platinum-wire nanocages, PtC nanotips, and self-assembled nanobubbles on gold films [32,33,34,35,36]. However, the most widespread uses of FIB-SEM in the field of metallic materials remain TEM specimen preparation, APT specimen preparation, and in situ compression experiments. These preparation techniques and the progress in their related analytical methods are introduced in detail in this section.

### 4.1. TEM Specimen Preparation

**Figure 6:**
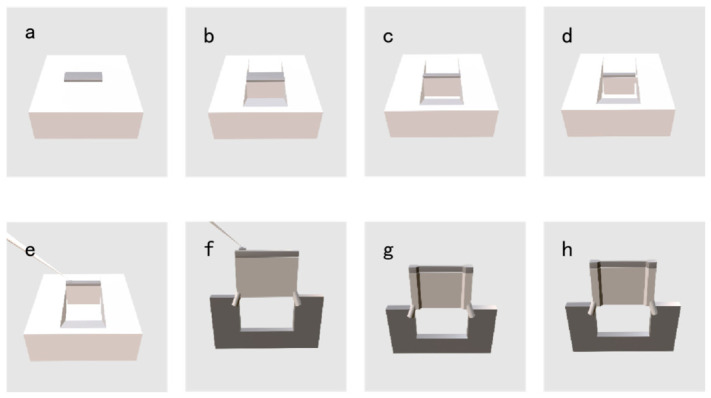
Three-dimensional schematic of TEM specimen preparation: (**a**) deposition; (**b**) coarse milling; (**c**) fine milling; (**d**) U-cutting; (**e**) lift-out; (**f**) welding; (**g**) final thinning; (**h**) cleaning.

TEM enables the nano/atomic-scale microstructural analysis of metallic materials. Under real-time SEM observation, FIB-SEM can be used to select regions of interest—such as crack tips, inclusions, and grain boundaries—and extract TEM specimens in either perpendicular or parallel orientations (lift-out). Taking the perpendicular lift-out method as an example, the procedure consists of eight steps: deposition, rough cutting, fine cutting, U-cutting, extraction, welding, final thinning, and cleaning. Steps 1 through 7 (deposition to final thinning) are typically performed at a machining voltage of 10–30 kV, while the cleaning step is typically carried out at 2–5 kV. The range of cleaning voltages appears to be relatively broad for the observation of surface morphologies and may be suitable for the detection of material defects. A schematic diagram of the perpendicular lift-out TEM specimen preparation process is shown in Figure 6.

This technique continues to be updated and extended. Qiao et al. [37,38] proposed a multi-voltage FIB-SEM lift-out technique for extracting cross-sectional TEM specimens from diamond and Zr alloys. TOMUS et al. [39], JUBLOT et al. [40], and WANG Xueli et al. [41] successfully developed FIB-SEM lift-out techniques for preparing plan-view TEM specimens suitable for crack analysis. Deng et al. [42] proposed a thin-film-embedding-strengthened FIB (FeS-FIB) technique, based on FIB-SEM, to successfully extract TEM specimens from both spherical microparticles and nanosheet-type structures, and they successfully analyzed the lattice structures of Ni and Fe double hydroxide (Ni,Fe-LDH) nanosheets.

### 4.2. APT Specimen Preparation

**Figure 7:**
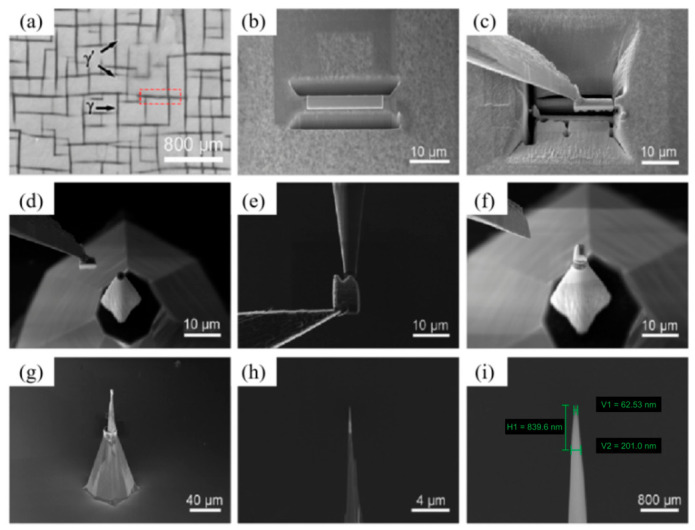
Standard lift-out procedure for FIB-prepared APT specimens (adopted from Ref. [70]): (**a**) Pt deposition; (**b**) coarse milling; (**c**) fine milling; (**d**) U-cutting; (**e**) lift-out; (**f**,**g**) welding; (**h**) final thinning; (**i**) cleaning.

APT enables the three-dimensional atomic-scale chemical compositional analysis of metallic materials. Under real-time SEM observation, FIB-SEM can be used to select regions of interest—including precipitate phases [43,44,45,46,47,48,49,50], grain boundaries [51,52,53,54,55,56,57,58,59], phase interfaces [60,61,62], and other regions of interest [63,64,65]—and extract APT specimens in either perpendicular or parallel lift-out orientations. MILLER et al. [66,67] adapted the TEM specimen preparation concept to propose the lift-out method for APT, which involves Pt protection, cut-and-extract, welding to microstubs, and final annular milling to achieve the precise targeting of regions of interest. After the continuous refinement of the technical details by subsequent researchers [68,69], the lift-out method has become the most universal, standard approach to APT specimen preparation. A schematic diagram of the perpendicular APT lift-out specimen preparation process is shown in Figure 7 [70].

These methods have been widely applied to metallic materials. Takahashi et al. [63] used APT analysis to investigate micron-scale spherical oxide inclusions in titanium-containing weld metal, revealing non-uniformly distributed multi-layered oxide shells of varying composition formed on an oxide core; the outermost oxide layer was rich in titanium and was surrounded by a manganese-depleted zone that was approximately 30–40 nm thick. Lee, Y. et al. [57] employed atom probe tomography (APT) to determine the temporal evolution of the composition after heat treatment, in order to resolve the uncertainty in the Pt-Pd phase diagram, particularly the proposed miscibility gap. Woods et al. [71] built on this foundation to explore APT specimen preparation techniques for various metals, oxides, supported frozen liquids, and battery materials at low temperatures, successfully characterizing materials such as nickel-coated AAO membranes.

## 5. Other FIB-Based Correlative Characterization Techniques

Precise control of the crystallographic orientation is also an important requirement for the FIB-SEM micro/nanofabrication of metallic materials. GAO et al. [72] proposed an FIB-SEM-EBSD combined TEM specimen preparation technique to prepare TEM specimens of Widmanstätten structures in γ-TiAl alloys with specific zone axes, thereby successfully resolving the atomic structure of the special interface (Σ-11 CSL) between Widmanstätten and lamellar structures. COJOCARU-MIRÉDIN O et al. [73] proposed an FIB-SEM-EBSD-PPMS combined APT specimen preparation technique, enabling the correlative characterization of the crystal orientation, physical properties, and atomic-scale composition.

Time-of-flight secondary ion mass spectrometry (TOF-SIMS), which enables the ppm- to ppb-level detection of all elements (from hydrogen H to transuranium elements) at solid material surfaces, has attracted the attention of materials researchers and has been integrated with FIB-SEM. Shin-ichi et al. [74] demonstrated the application of FIB-SEM-TOF-SIMS in all-solid-state batteries (ASSBs) using a Bi ion source FIB-SEM system.

The mechanical behavior of metallic materials is also of major interest to researchers. UCHIC et al. [75] used micropillar compression to study the plastic flow behavior of pure nickel and superalloys at the micrometer scale. XUE et al. [76] proposed an FIB-SEM-TEM specimen preparation technique based on in situ SEM tensile testing, studying the microstructural evolution in the heat-affected zones (HAZs) of laser-welded DP1180 dual-phase steel joints. KIENER et al. [77] proposed a quantitative in situ TEM tensile testing technique using FIB-prepared nano-specimens to study the plastic deformation mechanisms of copper single crystals at the sub-micrometer scale (100–200 nm).

Beyond these established applications, emerging developments in automation, nanotomography, and machine learning are beginning to extend the capabilities of FIB-based characterization. Automated cryogenic FIB-SEM workflows have already been applied in biological specimen preparation, although comparable reports remain limited in metallic materials and lithium battery systems. For example, Casper et al. [78] used plasma-coupled ion sources to produce cryogenic lamellae of vitrified human cells in a robust and automated manner, with quality sufficient for pseudo-atomic structure determination and long cryogenic run times (> 1 week).

FIB-SEM nanotomography (FIB-SEM-NT) is another promising development. Cian et al. [79] utilized FIB-SEM nanotomography to investigate the morphologies of printed nanostructured networks at high resolutions. They reported 3D imaging with a voxel size of 5 nm × 5 nm × 15 nm and demonstrated a suite of techniques for extracting quantitative morphological information from these images. They also demonstrated a machine learning protocol to further enhance the resolution of FIB-SEM-NT-produced 3D volumes by generating intermediate network images and cubic voxels. Although this technique has already shown value in battery-related materials, its extension to metallic material studies, such as three-dimensional crystallographic analysis and the spatial distribution of dislocations, still requires further exploration.

Recent progress in atom probe tomography has also illustrated the growing role of data-driven analysis. Yue et al. [80] reported a surge in novel machine learning approaches in APT that aim to improve user independence and are efficient, reproducible, and statistically robust. By comparison, machine learning in FIB-SEM for metallic materials remains at a relatively early stage. In particular, further development is still needed in areas such as damage assessment, parameter optimization, automated workflow control, and data-driven analysis of complex microstructures. These directions may become important for the future evolution of FIB-SEM toward more intelligent and higher-throughput characterization.

## 6. Conclusions and Outlook

This paper systematically reviews the latest research advances in FIB-SEM technology in the field of metallic materials science. The principles and basic functions of FIB-SEM are introduced, together with detailed descriptions of its applications in morphological characterization and TEM and APT specimen preparation, as well as its integration with other characterization techniques. Currently, FIB technology is advancing along two parallel trajectories: the concurrent development of multiple-ion-source technologies (e.g., Ga^+^, Xe^+^, and other plasma sources) and their deep integration with multimodal characterization techniques. Looking forward, the integration of FIB-SEM with artificial intelligence and data-driven analysis may become an important direction for future development. At present, automated TEM sample preparation using FIB-SEM is still based on silicon (Si) protocols; however, for materials such as diamond and aluminum alloys, these Si-based preparation methods cause severe amorphization. These limitations also exist in areas such as image segmentation and three-dimensional reconstruction. Given the large number of relevant academic papers currently available, if a shared database of FIB-SEM processing parameters and material characterization results for multiple materials could be established and expanded, enhancing the breadth and depth of machine learning, it would be expected to effectively improve the intelligence of FIB-SEM technology.

## Figures and Tables

**Table 1:** Principles and performance characteristics of ion sources.

Parameter	LMIS	GFIS	Plasma
Principle	Field evaporation of liquid metal	Field ionization of gaseous atoms	Plasma generated by gas discharge
Ion Type	Metal ions (Ga^+^,Bi^+^)	Gas ions (He^+^, Ne^+^)	Gas ions (Xe^+^, Ar^+^)
Beam Spot Size	Small (1–5 nm)	Ultrafine (<0.5 nm)	Large (>10 nm)
Beam Current	Moderate (10^−3^–10^2^ nA)	Low (10^0^–10^2^ pA)	Very high (10^−2^–10^3^ nA)
Typical Applications	TEM/APT specimen preparation and nanoscale deposition	Ultra-high-resolution imaging and nanoscale patterning	High-throughput specimen preparation, deep etching and 3D tomography

## Data Availability

The original contributions presented in this study are included in the article. Further inquiries can be directed to the corresponding authors.
